# Fibrodysplasia Ossificans Progressiva (FOP): A Segmental Progeroid Syndrome

**DOI:** 10.3389/fendo.2019.00908

**Published:** 2020-01-10

**Authors:** Robert J. Pignolo, Haitao Wang, Frederick S. Kaplan

**Affiliations:** ^1^Department of Medicine, Mayo Clinic Alix School of Medicine, Rochester, MN, United States; ^2^Department of Physiology-Biomedical Engineering, Mayo Clinic Alix School of Medicine, Rochester, MN, United States; ^3^Kogod Center on Aging, Mayo Clinic Alix School of Medicine, Rochester, MN, United States; ^4^Department of Orthopaedic Surgery, University of Pennsylvania Perelman School of Medicine, Philadelphia, PA, United States; ^5^Department of Medicine, University of Pennsylvania Perelman School of Medicine, Philadelphia, PA, United States; ^6^Center for Research in FOP and Related Disorders, University of Pennsylvania Perelman School of Medicine, Philadelphia, PA, United States

**Keywords:** progeroid syndrome, fibrodysplasia ossificans progressiva, activin A, ACVR1, cell senescence

## Abstract

Segmental progeroid syndromes are commonly represented by genetic conditions which recapitulate aspects of physiological aging by similar, disparate, or unknown mechanisms. Fibrodysplasia ossificans progressiva (FOP) is a rare genetic disease caused by mutations in the gene for ACVR1/ALK2 encoding Activin A receptor type I/Activin-like kinase 2, a bone morphogenetic protein (BMP) type I receptor, and results in the formation of extra-skeletal ossification and a constellation of others features, many of which resemble accelerated aging. The median estimated lifespan of individuals with FOP is approximately 56 years of age. Characteristics of precocious aging in FOP include both those that are related to dysregulated BMP signaling as well as those secondary to early immobilization. Progeroid features that may primarily be associated with mutations in ACVR1 include osteoarthritis, hearing loss, alopecia, subcutaneous lipodystrophy, myelination defects, heightened inflammation, menstrual abnormalities, and perhaps nephrolithiasis. Progeroid features that may secondarily be related to immobilization from progressive heterotopic ossification include decreased vital capacity, osteoporosis, fractures, sarcopenia, and predisposition to respiratory infections. Some manifestations of precocious aging may be attributed to both primary and secondary effects of FOP. At the level of lesion formation in FOP, soft tissue injury resulting in hypoxia, cell damage, and inflammation may lead to the accumulation of senescent cells as in aged tissue. Production of Activin A, platelet-derived growth factor, metalloproteinases, interleukin 6, and other inflammatory cytokines as part of the senescence-associated secretory phenotype could conceivably mediate the initial signaling cascade that results in the intense fibroproliferative response as well as the tissue-resident stem cell reprogramming leading up to ectopic endochondral bone formation. Consideration of FOP as a segmental progeroid syndrome offers a unique perspective into potential mechanisms of normal aging and may also provide insight for identification of new targets for therapeutic interventions in FOP.

## Introduction

Aging may be a unique biological process, since evolutionarily there appears to be an absence of genes specifically selected to cause it (1, 2). Rather, age-related changes may be the unprogrammed results of optimization for early reproductive success. Thus, senescence at the organismal level represents a phenomenon with low mechanistic conservation among disparate metazoans and so mechanisms of human aging do not necessarily have metazoan counterparts in every situation. For example, replicative senescence or the loss of proliferative capacity in replication-competent somatic tissues is not a potential mechanism for aging in organisms where soma compartments are post-mitotic, such as *C. elegans* (3).

A complementary approach to studying a model system for aging in a lower organism is to directly study human aging. Although this would closely capture aspects of aging that are relevant to humans, it does not obviate consideration for the highly polygenic nature of age-related pathologies (4), the confounding effects of outbreeding, or environmental effects based on where and how individuals live. An approach to providing a scientifically tractable system, at least with respect to the former, is to study genetic diseases whose phenotypes mimic at least some (i.e., “segmental”) features of the usual human aging process (4, 5). Such segmental progeroid (i.e., premature or accelerated aging-like) syndromes are usually monogenic and may thus be simple enough to provide insights into the causes of their pathology. Studied within the context of theories for physiological aging, observations made in segmental progeroid syndromes may also explain certain aspects of normal aging. Despite being only partial phenocopies of normal aging (i.e., some tissues show aging features and other not), these segmental progeroid syndromes provide experimental tractability, with varying fidelity, that is the rationale for their use as paradigm for natural deteriorative changes that occur over time. Single-gene mutations that impact multiple aspects of the physiological aging phenotype may exert their action through developmental alterations that have consequences for post-maturational aging, and importantly, for regulation of the rates of post-maturational aging after normal development.

Here we propose that consideration of fibrodysplasia ossificans progressiva (FOP) as a segmental progeroid syndrome offers a unique perspective into potential mechanisms of normal aging and may also provide insight for identification of new targets for therapeutic interventions in FOP.

## Segmental Progeroid Syndromes as a Model to Investigate Human Aging

Representative segmental progeroid syndromes are shown in Table 1. Several are monogenic or at least affect the same or similar pathways when more than one gene causes the same phenotype within the same syndrome. The putative mechanism(s) by which aging phenotypes are manifested are similar in several syndromes, including decreased genome maintenance and accelerated cellular senescence. All of the syndromes reduce mean lifespan or life expectancy.

**Table 1:** Representative segmental progeroid syndromes including FOP (4–7).

**Syndrome**	**Inheritance**	**Approximate mean life-span (years)**	**Causative mutation**	**Possible mechanistic relevance to natural aging**
Down	*De novo* trisomy	60	Many genes involved in phenotype^a^	Decreased genome maintenance
Werner	Autosomal recessive	47	WRN	Decreased genome maintenance; altered DNA damage responses; accelerated cell senescence
Dyskeratosis congenita^b^	X-linked; autosomal dominant	Variable^c^	DKC1; TERC	Accelerated cell senescence
Cockayne	Autosomal recessive	20	CS-A (ERCC8); CS-8 (ERCC6)	Decreased genome maintenance
Hutchinson-Gilford	Dominant negative	12	LMNA	Altered DNA damage responses; accelerated cell senescence
Ataxia telangiectasia	Autosomal recessive	20	ATM	Decreased genome maintenance; Accelerated neurodegeneration; Reduced immune diversity
Berardinelli-Seip^d^	Autosomal recessive	40	AGPAT2; BSCL2	Altered insulin signaling; decreased membrane integrity; increased glycation damage
Fibrodysplasia ossifcans progressiva	Sporadic; autosomal dominant	56^e^	ACVR1 (ALK2)	Injury-induced senescence; overactive activin A-BMP pathway signaling

a *For examples, GATA1, JAK2, DSCR1, DYRK1A*.

b *Information shown for the two most common forms*.

c *Life expectancy ranges from infancy to 60s*.

d *Congenital generalized lipodystrophy type 1 and 2*.

e *Estimated median life expectation*.

In the case of FOP, possible mechanisms for generation of an accelerated aging phenotype include injury-induced senescence and overactive activin A signaling. In comparison to other segmental progeroid syndromes, FOP represents an opportunity to study two different mechanisms by which aging phenotypes may be produced. Injury-induced senescence, especially in soft tissue such as muscle, has recently been described (8, 9) and muscle injury is a known cause of episodic inflammatory exacerbations or flare-ups in FOP (10, 11). Increased signaling through the bone morphogenetic protein (BMP) pathway, especially by activin A, has been implicated in osteoarthritis, sarcopenia, neurodegeneration, and other features associated with aging and FOP (discussed below under section Segmental Progeroid Features of FOP). Furthermore, activin A is a component of the senescence-associated secretory phenotype (SASP) (12, 13) and in FOP the mutated ACVR1/ALK2 encoding Activin A receptor type I/Activin-like kinase 2 (ACVR1/ALK2), a BMP type I receptor, is exquisitely sensitive to increased levels (14). Thus, injury-induced senescence leading to increased production of activin A may precipitate flare-ups in FOP and increased BMP signaling through activating mutations in ACVR1 may contribute to accelerated age-related changes in certain tissues. To test this hypothesis it will be necessary to examine the senescent cell burden in FOP lesion formation using markers of senescence in both patient samples and mouse models of FOP as well as analysis of the SASP in mouse models of FOP.

## Fibrodysplasia Ossificans Progressiva (FOP)

FOP is a strongly debilitating genetic disorder with hallmark features of congenital first toe malformations, progressive heterotopic ossification (HO) that produces normal bone at extra-skeletal locations, and accelerated features of aging (10, 11). The worldwide prevalence is 1/1,300,000–1/2,000,000 (15, 16). There is no ethnic, racial, gender, or geographic predilection to FOP. Early in life, episodic bouts of inflammatory soft tissue protuberances (i.e., exacerbations or flare-ups) develop which are often caused by injury, intramuscular injections, viral infections, muscular overuse, or fatigue (17, 18). These exacerbations convert connective tissues, including skeletal muscle, into HO. Tendons, ligaments, fascia, and aponeuroses are also affected, and together with transformed muscle, result in joint ankyloses and immobility. Atypical forms of FOP have been reported (19). Approximately 97% of patients with FOP harbor an activating mutation (617G > A; R206H) in ACVR1/ALK2 (6). Individuals with FOP variants also have heterozygous ACVR1 missense mutations in conserved amino acids. FOP is diagnosed clinically, with confirmation by genetic testing if available. The majority of FOP cases are sporadic (i.e., non-inherited mutations), but a small number of cases demonstrate germline transmission with inheritance in an autosomal dominant fashion (6). Although progressive HO is a hallmark feature, changes in early adulthood reminiscent of premature aging are evident.

Currently, there are no curative interventions, and the mainstay of treatment is focused on symptomatic relief using brief courses of high-dose corticosteroids for flare-ups, which may help to reduce the intense pain and edema associated with the early stages of ectopic bony lesion formation (10, 20). Steps to mitigate the likelihood of falls, decline in pulmonary function, and acquisition of viral infections are important prophylactic measures. The median life expectancy is about 56 years of age (7). Most patients require partial or complete assistance for ambulation by age 30, and common proximal causes of death include thoracic insufficiency syndrome and pneumonia (7). Factors contributing to the accelerated aging phenotype of FOP may be primarily related to ACVR1/ALK2 mutation, secondarily related to immobilization and disuse due to HO-associated joint ankyloses, or a combination of the two. Endpoints of current clinical trials focus on reducing heterotopic bone formation (20), but it is unclear if those therapies targeting mutant ACVR1/ALK2 signaling will also delay, prevent, or ameliorate the progeroid features of FOP. Furthermore, it is unclear if targeting the activin-A ligand, vs. the receptor or post-receptor pathways, will be sufficient to mitigate all aspects of the condition. Also, it is unknown if or how targeting activin-A and its signaling networks will impact its role in hypothalamic-pituitary-gonadal feedback.

## Segmental Progeroid Features of FOP

Progeroid features in FOP that may primarily be associated with mutations in ACVR1 include alopecia, subcutaneous lipodystrophy, hearing loss, myelination defects, osteoarthritis, heightened inflammation, menstrual abnormalities, and perhaps nephrolithiasis (Table 2).

**Table 2:** Progeroid features in FOP.

**System or tissue**	**Aging features in FOP**	**Characteristic(s) in FOP**
Skin	Alopecia; Subcutaneous lipodystrophy (21–24)	Alopecia seen in both sexes (19, 25–27); lipodystrophy may be associated with jaw ankylosis or recurrent flare-ups
Central nervous	Hearing loss; Myelination defects	Conductive and sensorineural hearing loss (19, 28–30); re-myelination deficits (31–33)
Respiratory	Decreased vital capacity; Pulmonary hypertension	Restrictive pulmonary function (7, 34)
Bone	Osteoporosis; Fractures	Osteoporosis (secondary) (35)
Muscle	Sarcopenia (36–38)	Sarcopenia of disuse is prominent
Joint	Osteoarthritis (19, 39, 40)	Often symmetrical
Immune	Inflammation; Predisposition to respiratory infections (7)	Acute inflammatory episodes (flare-ups) (10); chronic inflammatory state (41–45)
Reproductive	Menstrual abnormalities	Amenorrhea (19, 46, 47)
Renal	Nephrolithiasis (48)	Three times more likely compared to general population (49)

Alopecia is frequently observed in individuals with FOP and clinically is seen in both males and females. Evidence suggests that BMP signaling is involved in the control of the hair cycle (25). Increased BMP signaling through expression of BMP4, or its inhibition by the antagonist Noggin, causes progressive alopecia (26). In androgen-dependent alopecia, elevated BMP signaling in early (refractory) telogen likely mediates the retention of quiescent bulge stem cells (27). The case for elevated BMP signaling in lipodystrophy is less direct. Increased Fra-1 causes severe lipodystrophy (21) and both BMP-2 and TGF-β stimulate AP-1 activities, including the DNA binding activity of Fra-1 (22). Alternative explanations for subcutaneous lipodystrophy include decreased caloric intake after jaw ankylosis and the effects of recurrent inflammatory flare-ups. With respect to the latter, activation of the NF-κβ pathway during periods of acute or chronic inflammation may contribute to loss of subcutaneous fat. For example, activation of the NF-κβ pathway due to ubiquitination defects has been associated with lipodystrophy (23, 24).

Conductive and sensorineural hearing loss are common in FOP (28) and with prebycusis. Conductive hearing loss occurs when sound waves are not relayed efficiently to the inner ear, while sensorineural hearing loss is related to sensory organ (cochlea and associated structures) dysfunction or damage to the vestibulocochlear nerve (cranial nerve VIII). In humans, *NOGGIN* (*NOG*) gene mutations are associated with a few autosomal dominant conditions like proximal symphalangism and multiple synostoses which are characterized by skeletal defects and fusion of adjacent bones. Synostosis of one or more ossicles in the ear promotes conductive hearing loss. Proper formation of the skeleton requires balanced levels of BMPs and Noggin and the conductive hearing loss in *Nog*^+/−^mice results from an ectopic bridge of bone between the stapes and the tympanum, interfering with the normal mobility of the ossicle (29). BMP signaling is also required for inner ear development, including patterning of sensory regions in the cochlea that process sound (30). It is likely that hearing loss in FOP is due to increased BMP signaling very early in life affecting both/either the cochlear sensory regions and/or motion of ossicles. Later in life, synostosis of the ossicles due to HO may be the predominant cause of progressive hearing loss.

Demyelinated lesions and focal inflammatory changes of the CNS are seen in both mouse models of FOP and in CNS white matter lesions in FOP patients (31). BMP signaling is a potent inhibitor of oligodendroglial differentiation and remyelination (32), and gain-of-function mutations in ACVR1/ALK2 predictably enhance this potent inhibition. Dysregulated BMP signaling causes CNS demyelination, and CNS demyelination is one of the underlying mechanisms for the observed atypical neurologic phenotypes in FOP patients. With normal aging, decreased CNS remyelination becomes more prominent over time (33, 50). In addition, aging is associated with decreased hippocampal neurogenesis and concomitant hippocampus-dependent cognitive functions (51). There is an inverse relationship between CNS levels of BMP4 expression and noggin with age, with the former increasing substantially in the mouse dentate gyrus and the latter decreasing. This results in a profound elevation of phosphorylated-SMAD1/5/8, a key effector of BMP signaling. As with aging in mice, a large increase in BMP4 expression is seen in the dentate gyrus of older humans without known cognitive dysfunction (51). Increased BMP signaling is related to impairments in neurogenesis and to age-related cognitive changes (51) and aspects of these processes may be phenocopied in FOP.

Accelerated osteoarthritis is commonly found in FOP. Terminal differentiation of chondrocytes may be delayed or prevented by abrogation of BMP signaling in articular cartilage, and mitigation of this blockage or increased BMP signaling may then contribute to endochondral ossification and breakdown of cartilage matrix (39). In cartilage, TGFß and BMP are necessary for normal joint development and maintenance and their dysregulation has been associated with the pathogenesis of osteoarthritis. Interestingly, osteoarthritic patients have significantly higher serum levels of BMP-2 and BMP-4 compared to non-diseased humans and appear to characterize patients who have degenerative joint disease severe enough to require total joint replacement (40).

Heightened inflammation in FOP can be acute (as in episodic flare-ups) as well as chronic (as in an elevated pro-inflammatory state). The inflammatory nature of flare-ups in FOP is clinically obvious and well-described (41). In FOP patients without clinically evident HO, increased serum levels of cytokines, including IL3-, IL-7, IL-8, and IL-10, suggest a persistent pro-inflammatory state (42). So-called “inflammaging” refers to the chronic, sterile, low-grade inflammation which develops as part of normal aging, and is thought to contribute to the pathogenesis of multiple age-related diseases (43). In FOP, both acute and chronic inflammation may be related to the role of activin A in the initiation and persistence of the inflammatory response (44, 45).

Early menstrual abnormalities in FOP, including amenorrhea, are clinically recognized but have not been objectively studied or described. Roles for activin A in the ovulation cycle as well as in endometrial repair after menses have been reported and are perhaps causally related (46, 47).

Progeroid features in FOP that may secondarily be related to immobilization from progressive HO include decreased vital capacity, osteoporosis, fractures, sarcopenia, and predisposition to respiratory infections (Table 2). These manifestations represent an opportunity to study the contribution of disuse to the normal aging phenotype typified by the decreased physical activity, sedentary predilection, and increased likelihood of prolonged bed rest in older adults. If physiological aging is the result of primary aging processes interacting with or superimposed upon the pathophysiological consequences of inactivity (36), then specific characteristics of precocious aging in FOP due to disuse would be amenable to study in isolation. As an illustration, unloading of the normotopic skeleton due to bridging heterotopic bone results in osteoporosis. Another example is the increase in chest wall rigidity and decreases in elastic recoil and force-generating capacity of respiratory muscles that contribute to diminished vital capacity and predisposition to respiratory infection.

Some manifestations of precocious aging may be attributed to both primary and secondary effects of FOP. Sarcopenia in FOP likely represents the effects of both disuse atrophy due to joint ankyloses as well as increased activin A signaling causing both increased muscle catabolism and inhibition of myoblast differentiation (37, 38). Nephrolithiasis in FOP could be related to inadequate fluid intake due to functional difficulties in voiding, immobilization itself, and the effects of activin A on kidney function (48, 49).

## Injury, Reprogramming *in vivo*, and Cellular Senescence

**Figure 1:**
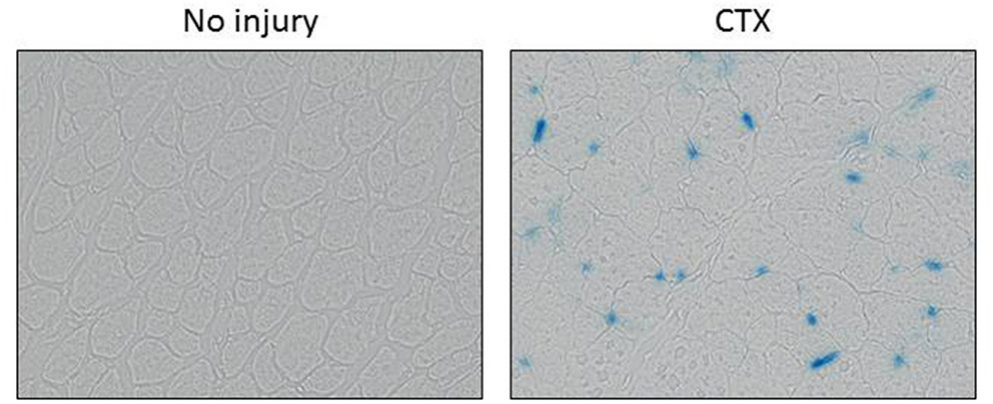
Muscle injury-induced senescence. Senescence-associated β-galactosidase (SAβ-gal) staining of the tibialis anterior muscle of a wild-type mouse is shown without injury **(left)** and 5 days after injury via cardiotoxin (CTX) injection. SAβ-gal-stained cells appear blue. Images are courtesy of Haitao Wang, Ph.D., Mayo Clinic, Rochester, MN, USA).

Injury, in general, is associated with accumulation of senescent cells [see (8) and Figure 1]. Growing evidence suggests that injury-induced reprogramming in skeletal muscle is facilitated by the accumulation of senescent cells at or near the site of damaged tissue (8). Bothe acute and chronic injury enables transcription-factor-mediated reprogramming in damaged muscle (8). The reprogramming effect of senescence appears to be due to the release of interleukin 6 (IL-6) and perhaps other components of the senescence-associated secretory phenotype (SASP) (8, 52). Senescence and the SASP facilitate the reprogramming of neighboring non-senescent cells but also recruit macrophages for the removal of necrotic tissue (8).

Paracrine release of IL-6 and other factors secreted by senescent cells promote reprogramming by Oct4, Sox2, Klf4, and c-Myc (OSKM) in non-senescent cells (53) into pluripotent cells (also called induced pluripotent stem cells or iPSCs). A direct relationship has been demonstrated between senescence and OSKM-driven reprogramming. In cells lacking p16INK4a/ARF (i.e., cells that do not undergo senescence), their ability to reprogram is severely compromised (9, 53). Furthermore, pharmacological inhibition of NF*k*B, a major driver of cytokine production and the SASP, reduces *in vivo* reprogramming (9). Aging, which is associated with higher levels of cellular senescence, also favors OSKM-driven reprogramming. Similarly, in physiological conditions of wound healing, senescence triggered by injury could promote cell dedifferentiation to mediate repair of damaged tissue (9, 53).

## Roles of Cellular Senescence in FOP Lesion Formation

**Figure 2:**
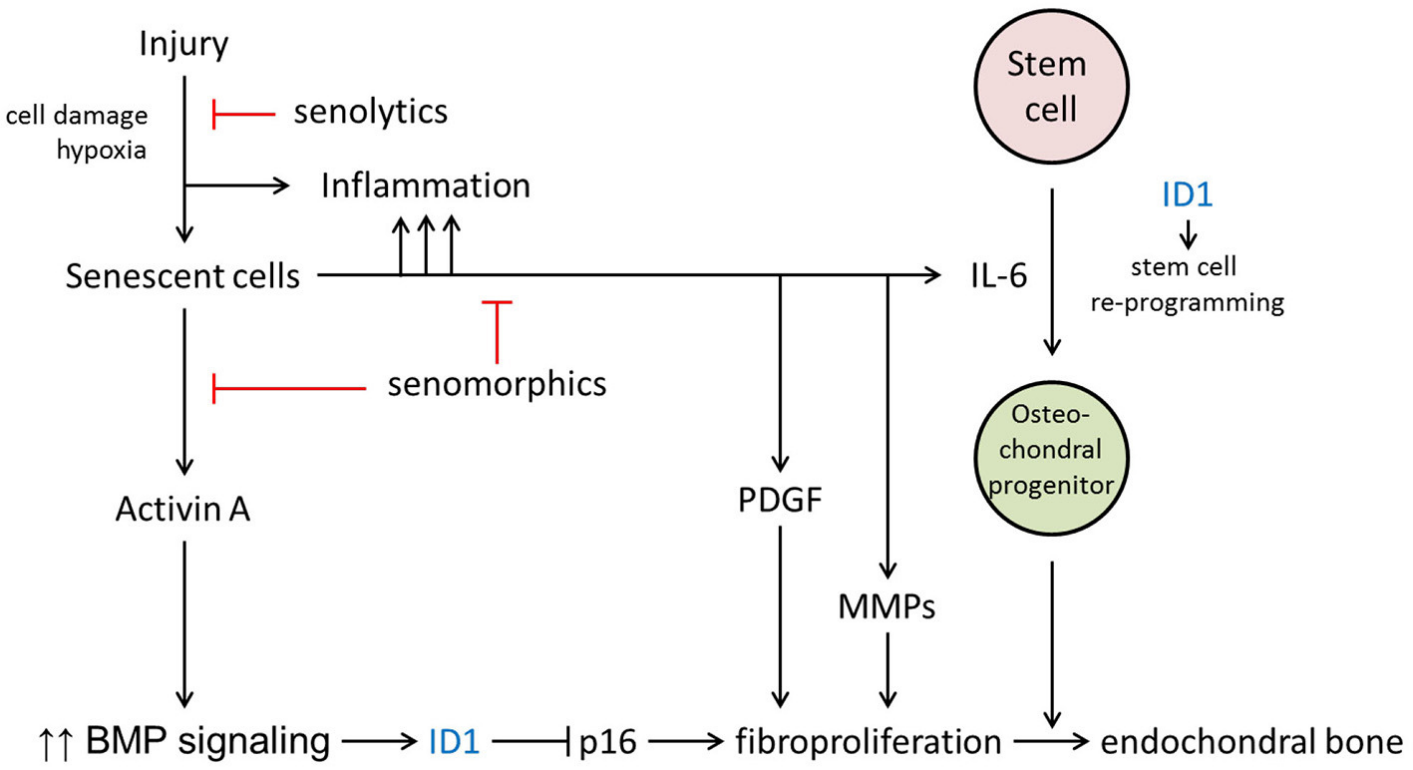
Potential roles for cellular senescence in FOP lesion formation. The major hypothesized contributions of senescence are through the production of activin A, IL-6, and other components of the SASP. _T_, inhibitory pathways;${\;}_{\text{T}}$, inhibitory action of senotherapeutic drugs.

In FOP, injuries due to soft tissue trauma, viral infection, muscular stretching, and even fatigue due to overuse can precipitate a flare-up. Tissue damage causes pathogen-associated molecular patterns (PAMPs) and damage-associated molecular patterns (DAMPs) in response to microbial and endogenous injury in the setting of a hypoxic microenvironment (54, 55). As the result of tissue injury, senescent cells accumulate and potentially contribute to early events that enhance BMP signaling and facilitate the reprogramming of tissue-resident stem cells (Figure 2).

Senescence is a cellular response to damage characterized by an irreversible cell cycle arrest and then by the SASP (56, 57). The SASP produces at least two factors that can directly promote increased BMP signaling and stem cells reprogramming—activin A and IL-6, respectively (Figure 2). It is well-established that activin A stimulates BMP signaling in FOP cells, owing to the causative mutations in the ACVR1 gene. In addition to the permissive effects of IL-6 in reprogramming, FOP cells show an increased efficiency of iPSC generation (58). In normal cells, the generation of iPSCs is facilitated by transduction of mutant ACVR1 or SMAD1 or by the early addition of BMP4 during the reprogramming. ID genes, downstream targets of BMP-SMAD signaling, are important for iPSC generation and their signaling through this pathway can inhibit cell senescence due to p16/INK4A, which otherwise serves to prevent reprogramming (58). Thus, ID1 and other ID genes may serve to both enhance expansion of the FOP early lesion as well as stimulate production of osteochondral progenitor cells. Enhanced BMP signaling promotes a tremendous fibroproliferative response, perhaps further accelerated by secretion of platelet-derived growth factor (PDGF) and matrix metalloproteinases (MMPs) by the SASP (Figure 2). Osteochondral progenitor cells derived from reprogrammed stem cells ultimately contribute to the endochondral bone formation which is the hallmark of mature FOP lesions. Other events that may contribute to formation of heterotopic bone cannot be excluded (59). However, in mouse models it will be possible to demonstrate if senescence-mediated tissue reprogramming in FOP lesions shifts lineage determination from a myogenic to a chondrogenic fate after injury.

Senescent cells may play multiple roles in the formation of HO in FOP and drugs which target senescent cells and/or the SASP may be candidates for therapeutic interventions. Compounds which selectively clear senescent cells (so-called senolytics) were first described on the basis of targeting pro-survival networks in senescent cells (60). Compounds that reduce the SASP (i.e., senomodulators), including inhibitors of the JAK/STAT pathway that plays an important role in regulating cytokine production, reduce systemic and adipose tissue inflammation in old mice (61). Rapamycin, a senomodulator, may be of particular benefit in FOP, since it also reduces activin-A mediated mTOR signaling (62). Many senotherapeutic agents have been reported, are effective in delaying or alleviating multiple age-related conditions in pre-clinical models, and are now being evaluated in clinical trials (63, 64). Their potential use in FOP offers a novel therapeutic approach to injury-induced flare-ups in FOP which should be further explored. We propose that senescent cell clearance and/or reduction in the SASP will ameliorate HO formation in mouse models of FOP and can be translated for use in patients with FOP.

## Conclusions

Monogenic segmental progeroid syndromes are important models for studying aspects of physiological aging. Features of precocious aging in FOP include both those that are related to dysregulated BMP signaling as well as those secondary to early immobilization and disuse. At the level of lesion formation in FOP, soft tissue injury resulting in hypoxia, cell damage, and inflammation may result in the accumulation of senescent cells as in aged tissue. Production of Activin A, interleukin 6, and other inflammatory cytokines as part of the SASP could mediate the initial signaling cascade that results in intense fibrosis as well as tissue-resident stem cell reprogramming prior to ectopic endochondral bone formation. This proposal requires experimental validation, but is amendable to testing in animal models. Consideration of FOP as a segmental progeroid syndrome may offer a unique perspective into potential mechanisms of normal aging, may increase understanding of BMP signaling as related to bone homeostasis and repair, and may also provide insight for identification of new targets for therapeutic interventions in FOP such as use of senotherapeutic drugs now in phase 1 and phase 2 clinical trials for aging-related conditions.

## Author Contributions

RP conceived the work, with substantial contributions from HW and FK. RP drafted the initial manuscript with HW and FK revising it critically. RP, HW, and FK gave final approval to the work and agreed to be accountable for all aspects.

### Conflict of Interest

The authors declare that the research was conducted in the absence of any commercial or financial relationships that could be construed as a potential conflict of interest.
